# Prehospital cardiopulmonary resuscitation duration and neurological outcome after out-of-hospital cardiac arrest among children by location of arrest: a Nationwide cohort study

**DOI:** 10.1186/s13049-019-0658-7

**Published:** 2019-08-23

**Authors:** Haruka Shida, Tasuku Matsuyama, Kosuke Kiyohara, Tetsuhisa Kitamura, Takefumi Kishimori, Takeyuki Kiguchi, Chika Nishiyama, Daisuke Kobayashi, Satoe Okabayashi, Tomonari Shimamoto, Takashi Kawamura, Taku Iwami

**Affiliations:** 1 Department of Preventive Services, Kyoto University School of Public Health, Kyoto, Japan; 2 Department of Emergency Medicine, Kyoto Prefectural University of Medicine, Kyoto, Japan; 3 Department of Food Science, Otsuma Women’s University, Tokyo, Japan; 4 Division of Environmental Medicine and Population Sciences, Department of Social and Environmental Medicine, Graduate School of Medicine, Osaka University, Osaka, Japan; 5 Kyoto University Health Services, Kyoto, Japan; 6 Department of Critical Care Nursing, Kyoto University Graduate School of Human Health Science, Kyoto, Japan

**Keywords:** Cardiac arrest, Paediatric, Prehospital EMS CPR duration, Favourable neurological outcome

## Abstract

**Background:**

Little is known about the associations between the duration of prehospital cardiopulmonary resuscitation (CPR) by emergency medical services (EMS) and outcomes among paediatric patients with out-of-hospital cardiac arrests (OHCAs). We investigated these associations and the optimal prehospital EMS CPR duration by the location of arrests.

**Methods:**

We included paediatric patients aged 0–17 years with OHCAs before EMS arrival who were transported to medical institutions after resuscitation by bystanders or EMS personnel. We excluded paediatric OHCA patients for whom CPR was not performed, who had cardiac arrest after EMS arrival, whose EMS CPR duration were < 0 min or ≥120 min and who had cardiac arrest in healthcare facilities. Prehospital EMS CPR duration was defined as the time from CPR initiation by EMS personnel to the time of prehospital return of spontaneous circulation or to the time of hospital arrival. The primary outcome was 1-month survival with a favourable neurological outcome (cerebral performance category scale 1 or 2). Statistical analysis was performed with Mann-Whitney U tests for numerical variables and chi-squared test for categorical variables. Univariable and multivariable logistic regression analyses were applied to assess the association between prehospital EMS CPR duration and a favourable neurological outcome, and crude and adjusted odds ratios and their 95% confidence intervals were calculated.

**Results:**

The proportion of patients with a favourable neurological outcome was lower in residential locations than in public locations (2.3% [66/2865] vs 10.8% [113/1048]; *P* < .001). In both univariable and multivariable logistic regression analyses, the proportion of patients with a favourable neurological outcome decreased as prehospital EMS CPR duration increased, regardless of the location of arrests (*P* for trend <.001). However, some patients achieved a favourable neurological outcome after a prolonged prehospital EMS CPR duration (> 30 min) in both groups (1.4% [6/417] in residential locations and 0.6% [1/170] in public locations).

**Conclusions:**

A longer prehospital EMS CPR duration is independently associated with a lower proportion of patients with a favourable neurological outcome. The association between prehospital EMS CPR duration and neurological outcome differed significantly by location of arrests.

## Background

Out-of-hospital cardiac arrest (OHCA) is a critical public health problem in industrialised countries [1–3], and only 3% of patients with OHCA are paediatric patients; thus, paediatric OHCA is rare compared to OHCA in adults [4, 5]. Paediatric cardiac arrests occur because of various factors, and numerous previous studies have investigated the association between several prehospital factors such as age and first documented rhythm and survival after paediatric OHCAs [4–9].

Paediatric OHCAs have a significant negative effect on society, especially in terms of the emotional burden of family members, and they have vital implications for medical staff; however, the medical challenges for paediatric OHCA patients have not been sufficiently discussed. Thus, the optimal cardiopulmonary resuscitation (CPR) duration for paediatric OHCA patients is difficult to determine. Although the paediatric CPR guidelines recommend that the decision to terminate CPR must not be based only on CPR duration [10], further evidence is needed to establish the appropriate paediatric CPR duration.

Previous studies demonstrated that bystander CPR and shocks by public-access automated external defibrillators (AEDs) are effective in improving the outcomes of paediatric OHCA patients [4, 11–13] and that differences in survival between locations among OHCA patients exist [14–16]. Thus, the location where the arrest occurs plays a role in prehospital CPR duration. The All-Japan Utstein Registry, which is a prospective nationwide, population-based registry of OHCA patients in Japan [17, 18], was launched to obtain information including OHCA locations since 2013 and has recorded approximately 4000 paediatric OHCAs that occurred before emergency medical services (EMS) arrival between 2013 and 2015.

This study aimed to investigate the relationship between the duration of prehospital CPR by EMS and 1-month survival with a favourable neurological outcome in paediatric OHCAs by location of arrests.

## Methods

### Study design, population, and settings

Details of the All-Japan Utstein Registry of the Fire and Disaster Management Agency (FDMA) of Japan have been previously described [17, 18]. This is a population-based observational study using a prospective, nationwide OHCA registry based on the international Utstein Style [19–21]. From this national registry, we extracted information on paediatric patients aged 0–17 years with OHCA before EMS arrival, who were resuscitated by bystanders or EMS personnel and were subsequently transported to medical institutions from 1 January 2013 to 31 December 2015. This study excluded (1) paediatric OHCA patients for whom CPR was not performed, (2) who had cardiac arrest after EMS arrival, (3) whose prehospital EMS CPR duration were < 0 min or ≥120 min and (4) who had cardiac arrest in healthcare facilities. The Ethics Committee of Kyoto Prefectural University of Medicine approved this study. The requirement of written informed consent was waived.

Cardiac arrest was defined as the cessation of cardiac mechanical activity, as evidenced by the absence of circulation signs and as confirmed by EMS personnel [19–21]. The aetiology of cardiac arrest was presumed to be medical in origin, unless the cardiac arrest was due to trauma, drug overdose, drowning, electrocution, or asphyxia, according to the current Utstein Style template [21]. The aetiologies were determined clinically by the physicians in collaboration with EMS personnel.

### EMS Systems in Japan

The Japanese paediatric population (aged 0–17 years) was approximately 20 million in 2015 [22], covering a geographic area of approximately 378,000 km^2^. EMS is provided by regional governments, and there were 750 fire stations with dispatch centres in 2015 [23]. Emergency life-saving technicians (ELSTs), who are highly trained emergency care providers, are allowed to start an intravenous line, provide an adjunct airway, and use semi-automated external defibrillators for OHCA patients. Specially trained ELSTs are allowed to intubate and administer adrenaline. Basically, each ambulance has a crew of three emergency providers, including at least one ELST. Cardiac arrest treatments were based on the Japanese CPR guidelines [24]. EMS providers are not permitted to terminate resuscitation in the field, excluding victims of decapitation, incineration, decomposition, rigor mortis, or dependent cyanosis. Thus, most OHCA patients treated by EMS personnel are transported to a hospital and included in the registry.

### Data collection and quality control

Data on resuscitation-related factors were prospectively obtained, including date, sex, age, cause of arrest, first documented rhythm, witness of cardiac arrest, time course of resuscitation, bystander CPR, dispatcher instruction, defibrillations by public-access AEDs, epinephrine administration, advanced airway management, prehospital return of spontaneous circulation (ROSC), 1-month survival, and neurological status 1 month after the event. When bystanders provided shocks using a public-access AED, the patients’ first documented rhythm was regarded as ventricular fibrillation (VF). In addition to the previous items of the international Utstein Style [19–21], the FDMA has started collecting detailed information on the location of OHCA occurrence since January 2013. According to the current Utstein Style template, locations of arrest are classified as follows: homes/residences, public areas, workplaces, recreation/sports event areas, streets/highways, healthcare facilities (clinic/nursing home), educational institutions, and others [21].

All survivors were followed for up to 1 month after the OHCA event by the EMS providers in charge. The neurological outcome was determined by the physician responsible for the care of the patient by a follow-up interview 1 month after successful resuscitation using the cerebral performance category (CPC) scale: category 1, good cerebral performance; category 2, moderate cerebral disability; category 3, severe cerebral disability; category 4, coma or vegetative state; and category 5, death/brain death [19–21].

A data form was filled out by the EMS personnel in cooperation with the physician in charge, and data were stored in the registry system on the FDMA database server. Data were logically checked via the computer system and were confirmed by the FDMA. If the data form was incomplete, the FDMA returned it to the respective fire station to complete the data.

### Outcome measures

The primary outcome measure was 1-month survival with a favourable neurological outcome after OHCA, which is defined as CPC 1 or 2 [19–21]. The secondary outcome measures were prehospital ROSC and 1-month survival. ROSC was defined as the restoration of a sustained spontaneous perfusing rhythm [25].

### Statistical analysis

Based on previous studies, prehospital EMS CPR duration was defined as the time from CPR initiation by EMS personnel to the time of prehospital ROSC (in cases where prehospital ROSC was achieved) or to the time of hospital arrival [26–29], and prehospital EMS CPR duration was classified into seven categories: 0–5, 6–10, 11–15, 16–20, 21–25, 26–30, and ≥ 31 min. In this study, main analyses were performed separately by location of cardiac arrests (“residential locations” and “public locations”). We defined a “residential locations” as homes/residences and a “public locations” as either public arias, recreation/sports locations, street/highway, educational institutions, and other public places, using defined in the Utstein template [21].

Patient and EMS characteristics and their outcomes were compared between the groups using Mann-Whitney U tests for numerical variables and chi-squared test for categorical variables. Furthermore, univariable and multivariable logistic regression analyses were applied to assess the association between prehospital EMS CPR duration and a favourable neurological outcome, and crude and adjusted odds ratios (AORs) and their 95% confidence intervals (CIs) were calculated. As potential confounders, factors that were biologically essential and considered associated with clinical outcomes were included in the multivariable analysis based on previous studies [8, 9, 26, 27, 29, 30]. These potential confounding variables were age groups (0, 1–4, 5–12, and 13–17 years), sex (male, female), cause of arrest (medical, non-medical), witness of arrest (yes, no), dispatcher instruction (yes, no), first documented rhythm (VF/pulseless ventricular tachycardia [VT], pulseless electrical activity [PEA], asystole), bystander CPR (yes, no), shocks by public-access AED (yes, no), epinephrine administration (yes, no), and advanced airway management (yes, no). In addition, as a subgroup analysis, we assessed the differences of the patient and EMS characteristics and their outcomes according to specific public locations. We applied the logistic regression analyses, using the same confounders with the main analyses. Public locations were divided into five groups: public areas, recreation/sports locations, educational institutions, streets/highways, and other locations (e.g., rice fields, sea, mountain, and unknown). Moreover, bystander ROSC, which was defined as a prehospital EMS CPR duration of 0 min, was added to prehospital EMS CPR duration categories. All statistical analyses were performed using SPSS statistical package 24.0 J (IBM Corp., Armonk, NY, USA). All tests were two-tailed, and *P* values <.05 were considered statistically significant.

## Results

**Fig. 1:**
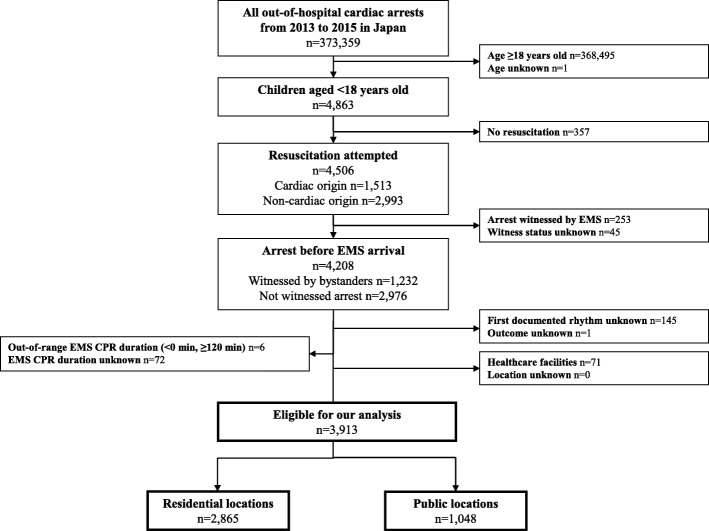
Patient flow of this study

During the study period, a total of 373,359 OHCA cases were registered, of which 3913 were paediatric OHCA patients and were analysed in this study [residential locations (2865, 73.2%) and public locations (1048, 26.8%)] (Fig. 1).

Patient and EMS characteristics and outcomes of paediatric OHCA patients according to the location of arrest are shown in Table 1. Those who collapsed in residential locations were more likely to be younger, had cardiac arrests of medical origin, and had dispatcher instructions and bystander CPR; moreover, they were less likely to have a witness of arrest, first documented shockable rhythm, and shocks by public-access AEDs. Nevertheless, no significant difference in the median of prehospital EMS CPR duration between the groups was found.

**Table 1:** Characteristics of out-of-hospital cardiac arrest among children by location of arrest

Characteristics	Total		Residential locations		Public locations		*P* value*
	(*N* = 3913)		(*N* = 2865)		(*N* = 1048)		
Weekday, n (%)	2756	(70.4)	2035	(71.0)	721	(68.8)	.175
Age, median (IQR)	2	(0–13)	1	(0–10)	9	(2–15)	<.001
Age group, n (%)							<.001
0 years old	1504	(38.4)	1320	(46.1)	184	(17.6)	
1–4 years old	749	(19.1)	582	(20.3)	167	(15.9)	
5–12 years old	640	(16.4)	374	(13.1)	266	(25.4)	
13–17 years old	1020	(26.1)	589	(20.6)	431	(41.1)	
Male sex, n (%)	2399	(61.3)	1668	(58.2)	731	(69.8)	<.001
Origin, n (%)							<.001
Medical	2725	(69.6)	2240	(78.2)	485	(46.3)	
Non-medical	1188	(30.4)	625	(21.8)	563	(53.7)	
Witnessed, n (%)	1065	(27.2)	528	(18.4)	537	(51.2)	<.001
Dispatcher instruction, n (%)	2497	(63.8)	2048	(71.5)	449	(42.8)	<.001
First documented rhythm, n (%)							<.001
VF/VT	245	(6.3)	62	(2.2)	183	(17.5)	
PEA	578	(14.8)	364	(12.7)	214	(20.4)	
Asystole	3090	(79.0)	2439	(85.1)	651	(62.1)	
Bystander CPR, n (%)	2340	(59.8)	1795	(62.7)	545	(52.0)	<.001
Shocks by public-access AEDs, n (%)	96	(2.5)	5	(0.2)	91	(8.7)	<.001
Epinephrine administration, n (%)	151	(3.9)	90	(3.1)	61	(5.8)	.001
Advanced airway management, n (%)	509	(13.0)	374	(13.1)	135	(12.9)	.887
EMS resuscitation times, median (IQR), min							
EMS response time (call to contact with the patient)	8	(7–10)	8	(7–10)	8	(7–11)	.020
Hospital arrival time (call to hospital arrival)	29	(23–37)	28	(23–36)	30	(24–39)	<.001
EMS CPR duration, median (IQR), min	19	(13–26)	19	(14–26)	19	(12–26)	.076
Prehospital ROSC, n (%)	273	(7.0)	124	(4.3)	149	(14.2)	<.001
One-month survival, n (%)	498	(12.7)	304	(10.6)	194	(18.5)	<.001
CPC 1 or 2, n (%)	179	(4.6)	66	(2.3)	113	(10.8)	<.001

*AED* automated external defibrillator, *CPC* cerebral performance category, *CPR* cardiopulmonary resuscitation, *EMS* emergency medical service, *IQR* first to third quartile, *PEA* pulseless electrical activity *ROSC* return of spontaneous resuscitation, *VF* ventricular fibrillation

*Comparisons between the two groups were evaluated with Mann-Whitney U tests for continuous variables and chi-square test for categorical variables

Regarding the primary outcome, the proportion of patients with a favourable neurological outcome was 2.3% (66/2865) in residential locations and 10.8% (113/1048) in public locations (*P* < .001; Table 1). Table 2 shows the ORs for prehospital EMS CPR duration and favourable neurological outcome. In both residential and public locations, the prehospital EMS CPR duration was significantly and independently associated with a favourable neurological outcome, and the proportion of patients with a favourable neurological outcome decreased as the prehospital EMS CPR duration increased (*P* for trend <.001). Moreover, the proportion of patients with a favourable neurological outcome in the group with a prehospital EMS CPR duration ≥31 min was significantly lower than that in the group with a prehospital EMS CPR duration of 0–5 min (58.7% [81/138] vs 1.2% [7/587], AOR 0.04, 95% CI 0.02–0.11 in all patients; 30.2% [16/53] vs 1.4 [6/417], AOR 0.07, 95% CI 0.02–0.22 in residential locations; and 76.5% [65/85] vs 0.6% [1/170], AOR 0.01, 95% CI 0.00–0.08).

**Table 2:** Odds ratios for cardiopulmonary resuscitation duration and 1-month survival with favourable neurological outcome by location of arrest

Outcome	All patients (*n* = 3913)						Residential locations (*n* = 2865)						Public locations (*n* = 1048)					
	n/N	%	Crude OR	(95% CI)	Adjusted OR	(95% CI)	n/N	%	Crude OR	(95% CI)	Adjusted OR	(95% CI)	n/N	%	Crude OR	(95% CI)	Adjusted OR	(95% CI)
EMS CPR duration																		
0–5 min	81/138	58.7	Ref	Ref	Ref	Ref	16/53	30.2	Ref	Ref	Ref	Ref	65/85	76.5	Ref	Ref	Ref	Ref
6–10 min	36/377	9.5	0.07	(0.05–0.12)	0.31	(0.16–0.61)	15/278	5.4	0.13	(0.06–0.29)	0.33	(0.12–0.88)	21/99	21.2	0.08	(0.04–0.17)	0.28	(0.10–0.76)
11–15 min	25/818	3.1	0.02	(0.01–0.04)	0.10	(0.05–0.20)	11/614	1.8	0.04	(0.02–0.10)	0.15	(0.06–0.40)	14/204	6.9	0.02	(0.01–0.05)	0.06	(0.02–0.17)
16–20 min	14/894	1.6	0.01	(0.01–0.02)	0.06	(0.03–0.12)	7/690	1.0	0.02	(0.01–0.06)	0.06	(0.02–0.18)	7/204	3.4	0.01	(0.00–0.03)	0.05	(0.02–0.16)
21–25 min	9/687	1.3	0.01	(0.00–0.02)	0.04	(0.02–0.10)	6/511	1.2	0.03	(0.01–0.07)	0.05	(0.02–0.17)	3/176	1.7	0.01	(0.00–0.02)	0.02	(0.01–0.10)
26–30 min	7/412	1.7	0.01	(0.01–0.03)	0.06	(0.02–0.14)	5/302	1.7	0.04	(0.01–0.11)	0.07	(0.02–0.25)	2/110	1.8	0.01	(0.00–0.03)	0.04	(0.01–0.20)
≥ 31 min	7/587	1.2	0.01	(0.00–0.02)	0.04	(0.02–0.11)	6/417	1.4	0.03	(0.01–0.09)	0.07	(0.02–0.22)	1/170	0.6	0.00	(0.00–0.01)	0.01	(0.00–0.08)
*P* for trend			<.001		<.001				<.001		<.001				<.001		<.001	

Adjusted for age category, sex, cause of arrest, witness of arrest, dispatcher instruction, first documented rhythm, bystander CPR, shocks by public-access AED, epinephrine administration, advanced airway management

*CPR* cardiopulmonary resuscitation, *EMS* emergency medical service, *OR* odds ratio, *CI* confidence interval

Table 3 shows the characteristics and outcomes of paediatric patients with OHCA in public locations. Those who had a cardiac arrest in recreation/sports locations and educational institutions were more likely to have bystander CPR, to be shocked using public-access AEDs, and to achieve prehospital ROSC. Hence, the median of prehospital EMS CPR duration was shorter, and the proportion of patients who had a favourable neurological outcome was higher. Table 4 shows the proportion of those with a favourable neurological outcome in public locations according to prehospital EMS CPR duration. In recreation/sports locations and educational institutions, the proportion of patients with a favourable neurological outcome in the groups with a short prehospital EMS CPR duration (< 15 min) was higher than that in other groups.

**Table 3:** Characteristics of out-of-hospital cardiac arrest among children by public locations of arrest

Characteristics	Public locations (total)		Public areas		Recreation/sports locations		Educational institutions		Streets/highways		Other locations	
	(*n* = 1048)		(*n* = 124)		(*n* = 49)		(*n* = 166)		(*n* = 311)		(*n* = 398)	
Witnessed, n (%)	537	(51.2)	63	(50.8)	21	(42.9)	109	(65.7)	226	(72.7)	118	(29.6)
Dispatcher instruction, n (%)	449	(42.8)	54	(43.5)	24	(49.0)	95	(57.2)	82	(26.4)	194	(48.7)
Bystander CPR, n (%)	545	(52.0)	51	(41.1)	38	(77.6)	139	(83.7)	103	(33.1)	214	(53.8)
Shocks by public-access AEDs, n (%)	91	(8.7)	2	(1.6)	10	(20.4)	72	(43.4)	3	(1.0)	4	(1.0)
Prehospital ROSC, n (%)	149	(14.2)	13	(10.5)	11	(22.4)	74	(44.6)	31	(10.0)	20	(5.0)
EMS CPR duration, median (IQR), min	19	(12–26)	17	(13–26)	15	(11–25)	12	(2–20)	20	(13–27)	21	(15–28)
One-month survival, n (%)	194	(18.5)	25	(20.2)	17	(34.7)	83	(50.0)	31	(10.0)	38	(9.5)
CPC 1 or 2, n (%)	113	(10.8)	11	(8.9)	11	(22.4)	68	(41.0)	13	(4.2)	10	(2.5)

*CPR* cardiopulmonary resuscitation, *AED* automated external defibrillator, *ROSC* return of spontaneous resuscitation, *CPC* cerebral performance category, *EMS* emergency medical service, *IQR* first to third quartile

**Table 4:** Cardiopulmonary resuscitation duration and 1-month survival with a favourable neurological outcome by public locations of arrest

Outcome	Public areas (*n* = 124)		Recreation /Sports locations (*n* = 49)		Educational institutions (*n* = 166)		Streets/highways (*n* = 311)		Other locations (*n* = 398)	
	n/N	%	n/N	%	n/N	%	n/N	%	n/N	%
EMS CPR duration										
Bystander ROSC	0/1	0.0	6/6	100	35/38	92.1	0/2	0.0	0/1	0.0
1–5 min	0/2	0.0	3/3	100	15/15	100	4/11	36.4	2/6	33.3
6–10 min	2/18	11.1	0/3	0.0	9/16	56.3	4/32	12.5	6/30	20.0
11–15 min	5/28	17.9	2/14	14.3	5/31	16.1	2/63	3.2	0/68	0.0
16–20 min	3/24	12.5	0/7	0.0	2/25	8.0	2/57	3.5	0/91	0.0
21–25 min	0/19	0.0	0/6	0.0	1/20	5.0	1/58	1.7	1/73	1.4
26–30 min	1/11	9.1	0/3	0.0	0/8	0.0	0/31	0.0	1/57	1.8
≥ 31 min	0/21	0.0	0/7	0.0	1/13	7.7	0/57	0.0	0/72	0.0

*CPR* cardiopulmonary resuscitation, *ROSC* return of spontaneous circulation, *EMS* emergency medical service

## Discussion

Based on a nationwide, population-based OHCA registry in Japan, we demonstrated that a longer prehospital EMS CPR duration is independently associated with a lower proportion of patients with a favourable neurological outcome 1 month after the OHCA event. Nonetheless, some paediatric patients with a prehospital EMS CPR duration of > 30 min survived with a better neurological outcome.

Regardless of the location of arrest, the outcomes deteriorated as prehospital EMS CPR duration increased. This finding was similar to those of previous studies [26, 29, 31], which could be explained by the poor CPR quality and long CPR interruption as the duration increased. Moreover, several previous studies demonstrated that the prehospital CPR duration of most paediatric patients who survived after OHCA was ≤15 min [32]. In our study, the proportion of patients with a favourable neurological outcome decreased markedly in the group with a prehospital EMS CPR duration of ≥15 min. Nevertheless, some paediatric patients with a prehospital EMS CPR duration of > 30 min survived with better neurological outcome, although a prehospital CPR duration of > 30 min was an indicator of a poor outcome [32]. This finding could be partially explained by the improvement in the “chain of survival,” such as dissemination of bystander CPR, an increase in the number of public-access AEDs, advanced prehospital care, and differences in patient characteristics [4, 12, 13, 18].

The proportion of paediatric patients with a favourable neurological outcome after OHCAs in public locations according to prehospital EMS CPR duration was higher than that in residential locations, which was almost consistent with the findings from previous studies [14–16]. Specifically, in public locations, 76.5% (65/85) of paediatric OHCA patients with a prehospital EMS CPR duration within 0–5 min had a favourable neurological outcome, which was a markedly high rate. Children with OHCA in public locations were more likely to have factors associated with a favourable neurological outcome, such as witness of arrest, first documented shockable rhythm, and shocks by a public-access AED, compared to those in residential locations [6, 8, 9, 12, 13, 26]. In Japan, public-access AEDs have been installed (over 600,000 devices in 2015) [33], and the Japan AED Project was launched in 2014 to further disseminate public-access defibrillation [34]; the project aims to encourage laypersons to learn how to administer CPR and use AEDs for OHCA patients via mass media and various medical associations across Japan. Thus, our results suggest that these various efforts in Japan led to achieving early ROSC and improving the neurological outcome of paediatric patients with OHCAs in public locations.

This study additionally investigated prehospital EMS CPR duration according to specific public locations and demonstrated that recreation/sports locations and educational institutions have a shorter prehospital EMS CPR duration and a better outcome. These public locations were more likely to have factors associated with a favourable neurological outcome, such as witness of arrest, bystander CPR, and shocks by a public-access AED compared to other public locations [4, 11–13]. In Japan, the statement “Aiming for zero deaths: Prevention of sudden cardiac death in schools and sport locations” published by the Japan Circulation Society [35, 36], CPR education, and widespread installation of AEDs in those locations are specific strategies that could result in a higher proportion of bystander interventions.

OHCAs in residential locations were more likely to have factors associated with a poor neurological outcome, such as age < 1 year old, no witness of arrest, and no first documented shockable rhythm [6, 8, 9, 12, 13, 26, 32], and the proportion of patients who had prehospital ROSC in residential locations was also lower in our study. Therefore, a longer prehospital EMS CPR duration for paediatric OHCA in residential locations seems futile. Moreover, in residential locations, the proportion of paediatric patients with a favourable neurological outcome in the group with a prehospital EMS CPR duration within 0–5 min was half of or less than half that in public locations. Nevertheless, there were some paediatric patients in residential locations with a prehospital EMS CPR duration of > 30 min who survived with a good neurological outcome. Therefore, for paediatric OHCAs in residential locations, investigating who would benefit from receiving a prolonged EMS CPR duration is necessary.

This study has several limitations. First, we could not analyse the effect of the total CPR duration, i.e., including prehospital bystander CPR time prior to EMS arrival and in-hospital CPR duration. Also, we could not account for no-CPR duration before the initiation of CPR. Second, although we assessed the impact of EMS CPR duration by each location, the situation before the initiation of EMS CPR may vary according to the actual location, even if cardiac arrests occur in the same category of location. For example, EMS arrival times may be much longer and there may be a smaller number of bystanders in rural areas. This study could not adjust for these factors. Third, we did not obtain information about bystander CPR quality and in-hospital treatments.

Finally, although we adjusted for covariates, the possibility remains of having some unmeasured confounding factors that could influence the results. Nevertheless, this was the first study to demonstrate the relationship between prehospital EMS CPR duration and 1-month survival with a favourable neurological outcome according to the location of arrest among paediatric OHCA patients. The results of this study could provide helpful clues for improving the treatment strategy for paediatric OHCAs.

## Conclusions

On the basis of a nationwide, population-based OHCA registry in Japan and regardless of the location of arrest, a longer prehospital EMS CPR duration for paediatric patients with OHCAs was independently associated with a lower proportion of patients having favourable neurological outcomes 1 month after the arrest. However, the association between prehospital EMS CPR duration and neurological outcome differed significantly according to the location of arrest.

## Data Availability

The data that support the findings of this study are available from the FDMA of Japan, but the availability of these data is restricted.

## Abbreviations

AEDs: Automated external defibrillators
AORs: Adjusted odds ratios
CIs: Confidence intervals
CPC: Cerebral performance category
CPR: Cardiopulmonary resuscitation
ELSTs: Emergency life-saving technicians
EMS: Emergency medical services
FDMA: Fire and Disaster Management Agency
OHCA: Out-of-hospital cardiac arrest
PEA: Pulseless electrical activity
ROSC: Return of spontaneous circulation
VF: Ventricular fibrillation
VT: Ventricular tachycardia
